# Stable reference genes for RT-qPCR analysis of gene expression in the *Musa acuminata*-*Pseudocercospora musae* interaction

**DOI:** 10.1038/s41598-019-51040-z

**Published:** 2019-10-10

**Authors:** Erica Cristina Silva Rego, Tatiana David Miranda Pinheiro, Jose Dijair Antonino, Gabriel Sergio Costa Alves, Michelle Guitton Cotta, Fernando Campos De Assis Fonseca, Robert Neil Gerard Miller

**Affiliations:** 1 Instituto de Ciências Biológicas, Departamento de Biologia Celular, Universidade de Brasília, Campus Universitário Darcy Ribeiro, 70910-900 Brasília, DF Brazil; 2 Departamento de Agronomia-Entomologia, Universidade Federal Rural de Pernambuco, Rua Dom Manoel de Medeiros s/n, Dois Irmãos, 52171-900 Recife, PE Brazil

**Keywords:** Reverse transcription polymerase chain reaction, Biotic

## Abstract

Leaf pathogens are limiting factors in banana (*Musa* spp.) production, with *Pseudocercospora* spp. responsible for the important Sigatoka disease complex. In order to investigate cellular processes and genes involved in host defence responses, quantitative real-time PCR (RT-qPCR) is an analytical technique for gene expression quantification. Reliable RT-qPCR data, however, requires that reference genes for normalization of mRNA levels in samples are validated under the conditions employed for expression analysis of target genes. We evaluated the stability of potential reference genes *ACT1*, *α-TUB*, *UBQ1*, *UBQ2*, *GAPDH*, *EF1α*, *APT* and *RAN*. Total RNA was extracted from leaf tissues of *Musa acuminata* genotypes Calcutta 4 (resistant) and Cavendish Grande Naine (susceptible), both subjected to *P*. *musae* infection. Expression stability was determined with NormFinder, BestKeeper, geNorm and RefFinder algorithms. *UBQ2* and *RAN* were the most stable across all *M*. *acuminata* samples, whereas when considering inoculated and non-inoculated leaf samples, *APT* and *UBQ2* were appropriate for normalization in Calcutta 4, with *RAN* and *α-TUB* most stable in Cavendish Grande Naine. This first study of reference genes for relative quantification of target gene expression in the *M*. *acuminata*-*P*. *musae* interaction will enable reliable analysis of gene expression in this pathosystem, benefiting elucidation of disease resistance mechanisms.

## Introduction

Banana (*Musa* spp.) is an important monocotyledonous fruit crop in over 100 tropical and sub-tropical countries, contributing significantly as the world’s most consumed fruit towards global food security and poverty alleviation.

Leaf pathogens are important limiting factors in global banana production, with reduced photosynthetic capacity causing negative impact on crop yield, especially in tropical growing regions^1^. Numerous leaf diseases are caused by fungal pathogens that include *Cladosporium musae*, *Cloridium musae*, *Cordana musae*, *Curvularia* sp., *Drechslera gigantean*, *Hendersonia toruloides*, *Helminthosporium* sp., *Phyllacora musicola*, *Phyllosticta* spp, and the Dothideomycete *Pseudocercospora* sp. responsible for the important Sigatoka disease complex in *Musa*. *Pseudocercospora fijiensis*, *Pseudocercospora musae* and *Pseudocercospora eumusae* cause necrotic leaf lesions that can reduce functional leaf area to such an extent that crop yields can be reduced by 20–50% in affected areas^2,3^.

The majority of commercial banana cultivars lack genetic resistance to pathogens and, in situations where disease pressure is high, are dependent upon integrated disease management strategies that employ programmed applications of systemic or protective fungicides. Such dependence on agrochemical disease control, however, can increase production costs, with a negative impact on the environment, and increased selective pressure for emergence of resistant strains in pathogen populations^4,5^.

In order to advance the genetic improvement and the development of resistant *Musa* cultivars, elucidation of the cellular mechanisms and identification of genes involved in resistance responses to foliar pathogens is of fundamental importance. For this, high-throughput next-generation sequencing approaches (NGS) are applicable for RNA sequencing (RNAseq) and transcriptome analysis. To date, a number of large scale studies have been conducted in *Musa* to further understanding of defense responses and genes involved in response to different phytopathogens and pests, such as *Fusarium oxysporum* f. sp. *cubense* tropical race 4^6–8^ and the root-knot nematode *Meloidogyne incognita*^9^. In relation to foliar pathogens, to date, Illumina-based RNAseq transcriptome profiling of the host-pathogen interaction has been conducted with the Sigatoka leaf spot pathogen *P*. *fijiensis*^10^, with a characterization of unigenes expressed during interaction with *P*. *musae* determined through 454 sequencing of cDNA^11^.

In order to accurately quantify and validate gene expression derived from *in silico* NGS data, the reverse transcription quantitative real-time polymerase chain reaction (RT-qPCR) is today recognized as the benchmark approach for such work, given its’ specificity, sensitivity and rapidity^12,13^. For this, however, RNA sample quality, cDNA preparation and dilution, and PCR sample preparation and reaction execution must be standardized. In accordance with the Minimum Information for Publication of Real-Time Quantitative PCR Experiments (MIQE) guidelines^14^, normalization of expression of each target gene to that of a reference gene is also required to correct data for variations in samples that occur during cDNA preparation, with reference genes also subject to the same effect of cDNA quality^15,16^. For such normalization, the employment of appropriate reference genes for each organism, tissue type and time point is therefore essential. The ideal characteristic of a reference gene is a stable expression for a tissue type, regardless of developmental stage or experimental treatment conditions^15,17^. Genes that are involved in basal cell activities in plants have been assumed to offer constitutive expression across tissues and independent of experimental conditions. Commonly employed genes have included actin and tubulin, given their role in the cell cytoskeleton, genes involved in protein synthesis (elongation factor) and protein degradation (ubiquitin), and genes involved in glucose metabolism (glyceraldeide-3-phosphate dehydrogenase). With such housekeeping genes, however, a stable transcript response is not necessarily guaranteed across different experimental conditions, as observed in numerous plant species^18–20^ and can lead to inaccuracies in results, masking true interpretation of gene expression^16,21^.

In order to facilitate the identification of stable reference genes, a number of mathematical algorithms have been developed, including the programs geNorm, NormFinder and BestKeeper^22–24^. These have been widely employed in reference gene development across monocotyledonous plant species, including, for example, rice^25^, maize^26^, sugarcane^27^ and Setaria^28^.

In the case of *Musa*, Chen and colleagues^29^ conducted a study towards validation of reference genes for use in Cavendish bananas, with focus on fruit and peel at different developmental stages, after hormone treatment, and following fruit exposure to abiotic and biotic stress^29^. Genes were also validated using pooled plant tissues comprising root, leaf, flower, peel and pulp. Similarly, Podevin *et al*.^30^ investigated the stability of reference genes for analysis of gene expression in leaf and meristem tissues across varieties grown *in vitro*, under greenhouse conditions and in leaf disc material^30^. Zhang *et al*.^31^ also identified stable reference genes for root tissues in cultivars during interaction with *Fusarium oxysporum* f. sp. *cubense* race 1 and race 4 strains^31^. These data all highlight the importance of selection of multiple reference genes for normalization in accord with plant genotype, tissues, development stage and specific experimental conditions.

In this study, the stability of eight candidate reference genes for transcript normalization in whole plant leaf tissues was analyzed in two *M*. *acuminata* genotypes, contrasting in resistance to the foliar pathogen *P*. *musae*. The reliability of the most and least stable genes was determined through normalization of the relative expression of the target gene encoding RuBisCO activase (*RCA*). The selected reference genes will enable accurate gene expression analysis in this important pathosystem.

## Results

### Analysis of primer specificity and efficiency

**Figure 1:**
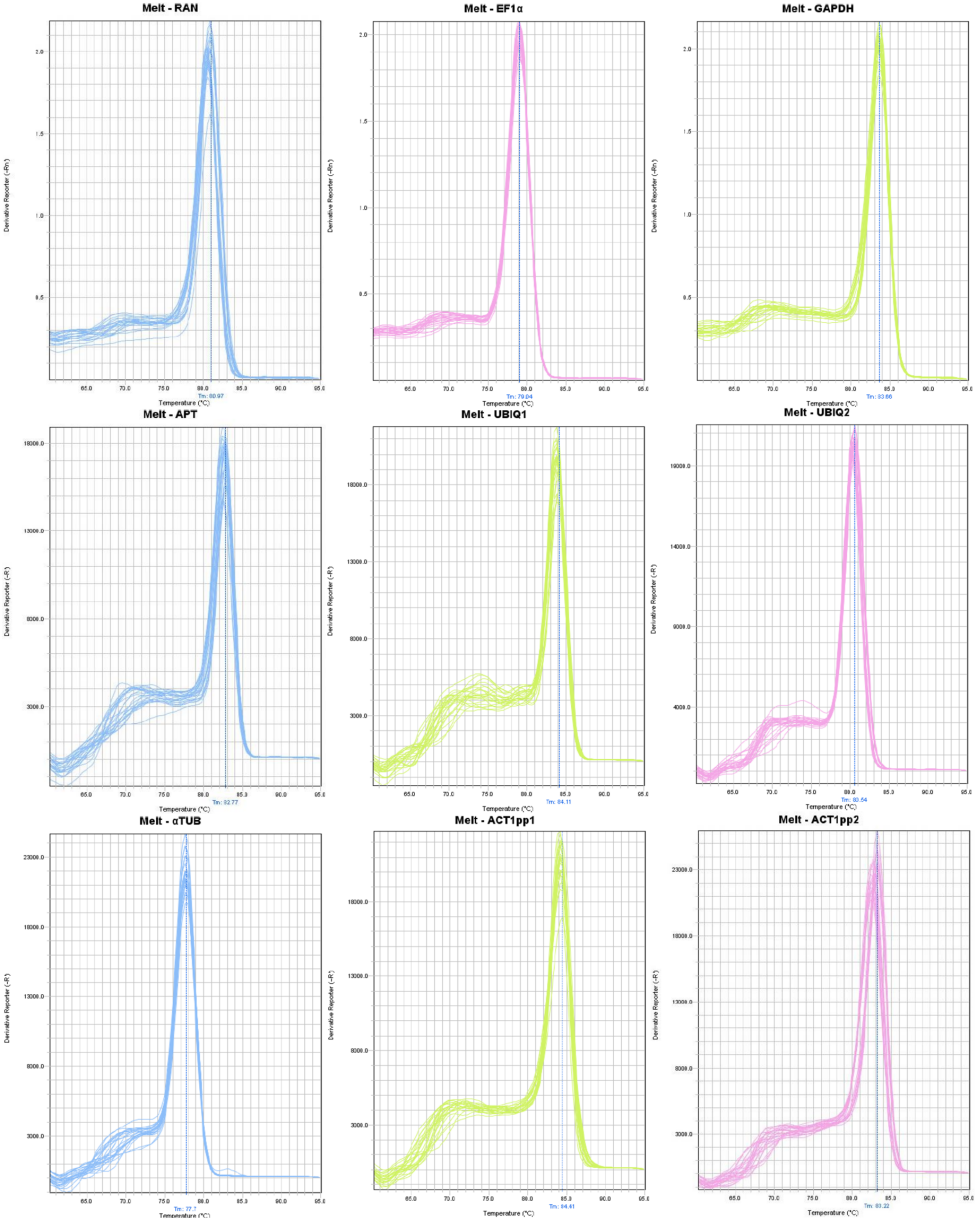
Dissociation curves of the candidate reference genes. Primers for genes (*ACT*) actin 1; (*APT*) adenine phosphoribosyltransferase; (*EF1α*) elongation factor 1 alpha; (*GAPDH*) glyceraldehyde-3-phosphate dehydrogenase; (*α-TUB*) alpha-tubulin; (*RAN*) GTP-binding nuclear protein; (*UBQ1*) ubiquitin 1 and (*UBQ2*) ubiquitin 2, revealing single peaks, each obtained from three technical replicates of different cDNA samples. No amplification was observed in negative controls lacking template cDNA.

A total of eight candidate genes were selected as potential reference genes in this study. Information on the candidate genes, in relation to accession number, primer sequences and amplification are summarized in Table 1. Specificity of each of the primers to a single gene locus was confirmed by dissociation curve analysis, with a single peak observed for each primer pair at the expected primer annealing temperature (Fig. 1), together with an absence of any signal in negative controls lacking template cDNA. PCR amplification efficiency values ranged from 90.55 to 98.21% across the candidate genes, as calculated by LinRegPCR software.

**Table 1:** Candidate reference genes and target gene for *Musa acuminata* leaf tissues, primer sequences and RT-qPCR efficiency.

Gene name	Description	Phytozome acession number	Primer name	Primer sequence (5′-3′)	Amplicon Tm (°C)	Amplicon size (bp)	PCR Amplification efficiency (%) ± SD
*ACT1*	Actin 1	GSMUA_Achr6G25350	Macu_Act1pp1-Fw1	CTGCGACAATGGTACTGGAAT	84.21	146	90.55 ± 0.022
			Macu_Act1pp1-Rv1	CCTCGTCACCAACATAAGCAT			
			Macu_Act1pp2-Fw2	GAGCGGAAGTACAGTGTCTGG	82.86	127	90.63 ± 0.019
			Macu_Act1pp2-Rv2	AGAAGCACTTCCTGTGGACAA			
*APT*	Adenine phosphoribosyltransferase	GSMUA_Achr2G18510	Macu_APT-Fw	TTGAACTGCCAGAATTGAAGG	82.7	125	98.21 ± 0.029
			Macu_APT-Rv	TTGGGAAGAACAGAGAAGCAG			
*EF1α*	Elongation factor 1 alpha	GSMUA_Achr10G22980	Macu_EF1a-Fw	GCTACAACCCAGAGAAGATACCCTT	78.87	80	96.63 ± 0.030
			Macu_EF1a-Rv	CAGGTTGGTAGACCTCTCAATCATG			
*GAPDH*	Glyceraldehyde-3-phosphate dehydrogenase	GSMUA_Achr11G11040	Macu_GAPDH-Fw	CATCAAGCAAGGACTGGAGAG	83.27	99	95.63 ± 0.027
			Macu_GAPDH-Rv	AAGCAGGGAGAACTTTTCCAA			
*α-TUB*	Alpha tubulin	GSMUA_Achr2G12390	Macu_TubA-Fw	GGAAGAAGTCGAAGCTTGGTT	77.65	95	92.96 ± 0.023
			Macu_TubA-Rv	GGAATGGGTGGATAGGACACT			
*RAN*	GTP-binding nuclear protein	GSMUA_Achr10G21070	Macu_RAN-Fw	AGCTGCAATTGGATCGAAAGT	80.58	90	97.70 ± 0.028
			Macu_RAN-Rv	GTAACATCGCCACCATAGCAT			
*UBQ1*	Ubiquitin 1	GSMUA_Achr7G04060	Macu_Ubq1-Fw	GGCAGGAGTAACGAACAACAA	83.14	142	92.59 ± 0.024
			Macu_Ubq1-Rv	CATTTCTCGTAGCTGGGTCAG			
*UBQ2*	Ubiquitin 2	GSMUA_Achr5G11110	Macu_Ubq2-Fw	AGAGAGATGCTGCAAAATCCA	80.48	140	94.44 ± 0.026
			Macu_Ubq2-Rv	CCAGCTGTCTGCTCTTGTTCT			
*RCA3*	Rubisco activase 3	GSMUA_Achr2T16450	MaqPCR-RCA3-Fw	GGGAAAGCAGCTCAACAGGT	85.80	107	93.4 ± 0.029
			MaqPCR-RCA3-Rv	TACAAGCAGCTCCCATCGTC

### Analysis of expression levels of candidate reference genes

**Figure 2:**
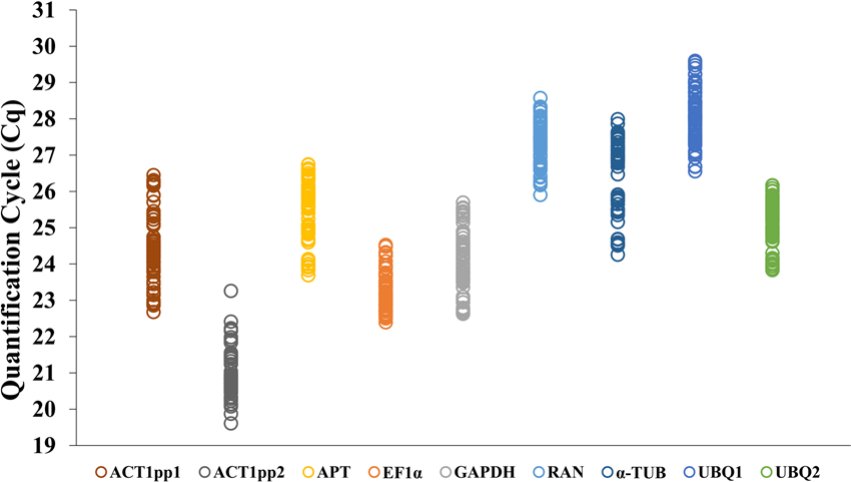
RT-qPCR quantification cycle (Cq) values for tested reference genes. Expression data is displayed as Cq values for each reference gene in leaf material from *Musa acuminata* genotypes Calcutta 4 and Cavendish Grande Naine, non-inoculated or inoculated with *Pseudocercospora musae*.

For each candidate reference gene, quantification cycle (Cq) values were presented for the expression data from each sample. Cq values ranged from 19.6 to 29.6, with broad differences observed between the candidate reference genes, highlighting the importance of statistical approaches for stability ranking in identification of accurate reference genes. The *ACT1* gene (amplified with primer pair 2 [pp2]) displayed the lowest Cq value, indicating that it was more expressed than the other candidate genes. Conversely, the *UBQ1* gene displayed the highest Cq values, indicating lowest gene expression. The Cq value ranges for the reference genes evaluated in the different *Musa* samples are displayed in Fig. 2, with a summary of all Cq values obtained for each treatment for the potential reference genes provided in Supplementary Table S1.

### Expression stability

The performance of the candidate reference genes was assessed in the four biological samples, divided into three experimental sets; global pooled *M*. *acuminata* Calcutta 4 and Cavendish Grande Naine leaf material (from non-inoculated and inoculated plants); pooled *M*. *acuminata* Calcutta 4 leaf material (from non-inoculated and inoculated plants); and pooled *M*. *acuminata* Cavendish Grande Naine leaf material (from non-inoculated and inoculated plants). Stability in expression and ranking of the eight candidate reference genes, amplified with nine primer pairs, was determined using mathematical algorithms geNorm, NormFinder and BestKeeper. Re-ranking of genes was conducted using RefFinder. Summarized results from all analyses are presented in Table 2.

**Table 2:** Expression stability analysis of each candidate reference gene for *Musa acuminata* leaf tissues based on algorithms geNorm, NormFinder and BestKeeper.

Sample	Gene	RefFinder		geNorm		NormFinder		BestKeeper	
		Rank	GM	Rank	M	Rank	SV	Rank	Std Dev [±Cp]
Global	*RAN*	3	2.28	2	0.33	4	0.014	3	0.493
	*EF1α*	2	1.86	4	0.479	3	0.014	1	0.442
	*GAPDH*	4	4.43	5	0.513	5	0.016	6	0.617
	*APT*	7	7	7	0.599	7	0.025	7	0.688
	*UBQ1*	6	5	3	0.371	1	0.005	5	0.589
	*UBQ2*	1	1.41	1	0.287	2	0.011	4	0.498
	*α-TUB*	8	8.24	8	0.639	8	0.030	9	0.915
	*ACT1pp1*	9	8.74	9	0.716	9	0.033	8	0.699
	*ACT1pp2*	5	4.56	6	0.543	6	0.022	2	0.450
Global C4	*RAN*	3	3.16	6	0.266	4	0.005	1	0.432
	*EF1α*	4	3.72	7	0.3	3	0.004	4	0.540
	*GAPDH*	8	7.71	9	0.355	9	0.009	9	0.751
	*APT*	1	1.19	1	0.087	5	0.005	2	0.525
	*UBQ1*	9	8.74	3	0.103	6	0.005	8	0.673
	*UBQ2*	2	1.86	2	0.091	1	0.003	3	0.529
	*α-TUB*	5	4.21	5	0.231	2	0.004	7	0.670
	*ACT1pp1*	7	6.93	8	0.325	7	0.007	6	0.654
	*ACT1pp2*	6	5.96	4	0.184	8	0.009	5	0.573
Global CAV	*RAN*	2	1.93	1	0.081	1	0.003	7	0.332
	*EF1α*	4	3.41	4	0.129	6	0.007	5	0.304
	*GAPDH*	5	4.47	2	0.097	2	0.003	4	0.302
	*APT*	7	5.45	7	0.266	7	0.012	3	0.270
	*UBQ1*	9	9	6	0.236	8	0.014	9	0.503
	*UBQ2*	3	3.13	5	0.18	4	0.004	6	0.309
	*α-TUB*	1	1.78	3	0.105	3	0.003	1	0.253
	*ACT1pp1*	8	8	9	0.363	9	0.016	8	0.495
	*ACT1pp2*	6	4.74	8	0.299	5	0.006	2	0.261

Overall re-ranking was performed using RefFinder. GM, Geometric Mean; M, Average expression stability; SV, Stability Value; Std Dev, Standard Deviation; Cp, Process Capability. With NormFinder and geNorm, low stability values indicate greater gene expression stability. With BestKeeper, genes with standard deviation values greater than 1 are considered as inconsistent.

**Figure 3:**
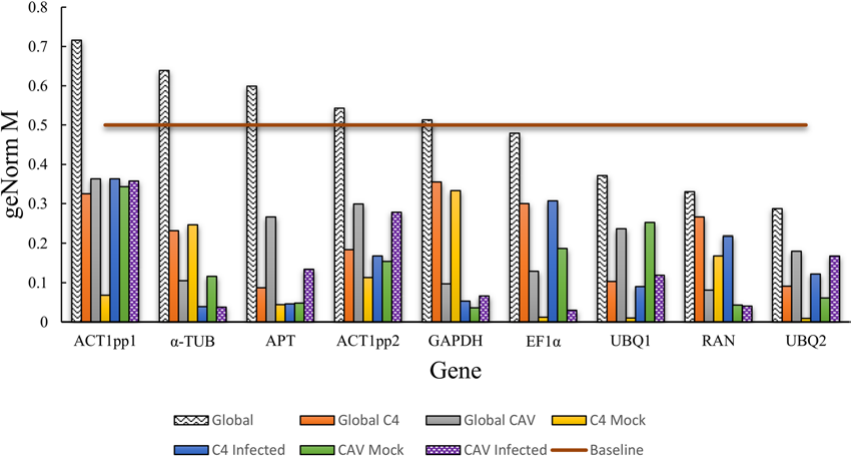
GeNormPlus-derived expression stability for each candidate reference gene for *Musa acuminata* leaf tissues. Values of M below a 0.5 cutoff indicate high stability rate.

On the basis of geNorm analysis, many candidate reference genes showed stability values below the default limit of 0.5 when employed on data for different *M*. *acuminata* samples (Table 2, Fig. 3). *UBQ2*, *RAN*, *UBQ1* and *EF1α* genes displayed stability values below 0.5 when employed on data for pooled *M*. *acuminata* Calcutta 4 and Cavendish Grande Naine leaf samples (from both non-inoculated and *P*. *musae*-inoculated, sample name ‘Global’), indicating greatest stability. In the case of samples from *M*. *acuminata* Calcutta 4 leaf material, either from non-inoculated or *P*. *musae*-inoculated plants (sample ‘Global C4’), all genes displayed stability values below the accepted threshold. *APT* and *UBQ2* were highlighted as the most stably expressed genes, followed by *UBQ1* and *ACT1pp2*. For samples from *M*. *acuminata* Cavendish Grande Naine leaf material (non-inoculated or *P*. *musae*-inoculated) (sample ‘Global CAV’), all genes once again displayed M-values below the accepted threshold, with *RAN* and *GAPDH* genes most stable, followed by *α-TUB* and *EF1α*.

**Figure 4:**
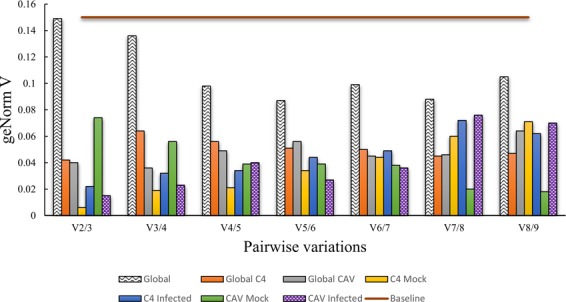
GeNormV-derived determination of the optimal number of reference genes for accurate RT-qPCR normalization of target gene expression. For data below a cutoff value of 0.15, the inclusion of additional reference genes will not contribute significantly to the normalization of gene expression data.

Calculation of pairwise variation was employed to determine the minimum number of reference genes required for stable normalization of gene expression data. According to Vandesompele and coworkers^22^, a conventional normalization of RT-qPCR based on a single reference gene can potentially lead to a more than 6-fold normalization error. Given this, geNorm automatically takes into account that at least two reference genes are employed for normalization. For all *M*. *acuminata* sample types (Global, pooled non-inoculated or *P*. *musae*-inoculated *M*. *acuminata* Calcutta 4 and Cavendish Grande Naine leaf samples; Global C4, pooled non-inoculated or *P*. *musae*-inoculated *M*. *acuminata* Calcutta 4 leaf samples; Global CAV, pooled non-inoculated or *P*. *musae*-inoculated *M*. *acuminata* Cavendish Grande Naine leaf samples; C4 mock, pooled non-inoculated *M*. *acuminata* Calcutta 4 leaf samples; C4 infected, *P*. *musae*-inoculated *M*. *acuminata* Calcutta 4 leaf samples; CAV mock, pooled non-inoculated *M*. *acuminata* Cavendish Grande Naine leaf samples; and CAV infected, *P*. *musae*-inoculated *M*. *acuminata* Cavendish Grande Naine leaf samples), the pairwise variation value of V2/3 was below the default cut-off value of 0.15, indicating that two reference genes are sufficient for accurate normalization of the gene expression data (Fig. 4). Values of V above the default limit, indicate that one or several extra reference genes would be required to improve RT-qPCR analysis^22^. Here, data revealed that the inclusion of further reference genes would not have a significant effect on accurate normalization of target gene expression.

NormFinder-based analysis provided gene stability ranking data with a certain overlap to that obtained with geNorm (Table 2). When considering the combined dataset ‘Global’, based on pooled *M*. *acuminata* Calcutta 4 and Cavendish Grande Naine leaf samples (non-inoculated or *P*. *musae*-inoculated), the four most stably expressed genes were in agreement, with *UBQ1*, *UBQ2*, *EF1α* and *RAN* genes ranked in order of greatest stability. In the case of data samples from *M*. *acuminata* Calcutta 4 leaf material, either from non-inoculated or *P*. *musae*-inoculated plants (sample ‘Global C4’), by contrast, only the *UBQ2* gene was highly ranked with both algorithms. In the case of analysis with the algorithm NormFinder, this gene ranked as the most stably expressed, followed by *α-TUB*, *EF1α* and *RAN*. When data was analyzed according to samples from *M*. *acuminata* Cavendish Grande Naine leaf material (non-inoculated or *P*. *musae*-inoculated) (sample ‘Global CAV’), three of the four most highly ranked stable genes were again in agreement with geNorm data, with *RAN* ranked as most stable, followed by *GAPDH*, *α-TUB* and *UBQ2*.

On the basis of results obtained following analysis with the program BestKeeper, all candidate genes were calculated to have a standard deviation value lower than 1 (Table 2). With this algorithm, a certain overlap in most stably expressed candidate reference genes was also observed when compared to ranking positions based on geNorm and NormFinder data. In the case of data considering the ‘Global’ dataset, three of the four most highly ranked stable genes were in agreement, with the *EF1α* gene ranked most stable, followed by *ACT1pp2*, *RAN* and *UBQ2*. When considering the data samples ‘Global C4’, three of the most highly ranked stable genes were in agreement with results obtained using NormFinder and two in agreement with geNorm-derived ranking. Here, the *RAN* gene ranked as most stable, followed by *APT*, *UBQ2* and *EF1α*. In the case of data derived from the samples ‘Global CAV’, two genes most highly ranked in terms of expression stability were in agreement with ranking data obtained using NormFinder and another two in agreement with ranking data obtained using geNorm. The four most highly ranked genes, in order of expression stability, were *α-TUB*, *ACT1pp2*, *APT* and *GAPDH*.

RefFinder was employed to enable a comprehensive re-ranking of expression stability in the candidate reference genes based on integration of the data from the previous three algorithms. When taking into account the complete ‘Global’ dataset, for both *M*. *acuminata* Calcutta 4 and *M*. *acuminata* Cavendish Grande Naine leaf material (non-inoculated and *P*. *musae*-inoculated), the four most highly ranked candidate reference genes, in order of stability, were: *UBQ2*, *EF1α*, *RAN* and *GAPDH*. In the case of the ‘Global C4’ dataset, the most stable candidate reference gene was *APT*, followed by *UBQ2*, *RAN* and *EF1α*. For the ‘Global CAV’ dataset, the most stable candidate reference gene was *α-TUB*, followed by *RAN*, *UBQ2* and *EF1α* (Table 2).

A summary of all candidate reference gene rankings with the mathematical algorithms is provided in Supplementary Table S2.

### Testing selected reference genes for normalization of *M*. *acuminata* genes expressed in leaves

**Figure 5:**
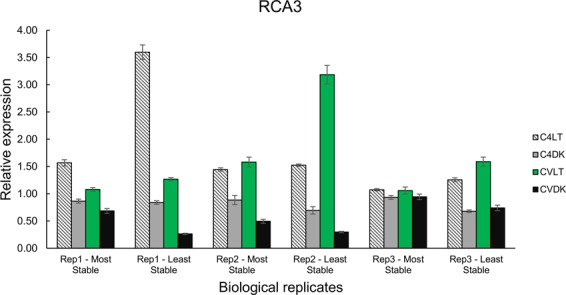
Relative expression levels of the RuBisCO activase gene *RCA3* in two *M*. *acuminata* genotypes using the most (*UBQ2* and *RAN*) and least (*α-TUB* and *ACT1pp1*) stable pair of reference genes. RT-qPCR was performed in three biological replicates of light and dark treated leaf samples. Calcutta 4 (C4), Cavendish Grande Naine (CV), Light (LT) and Dark (DK). Bars represent the standard error (±SE) calculated for three technical replicates.

For validation of reference gene ranking for leaf tissues, the relative expression of RuBisCO activase (*RCA*) was normalized against the reference genes. This catalytic chaperone is involved in modulating RuBisCO, a key enzyme in the plant photosynthetic pathway. Although three *RCA* genes were identified in the *M*. *acuminata* DH Pahang reference genome, only *RCA3* could be amplified by conventional PCR in both *M*. *acuminata* Calcutta 4 and *M*. *acuminata* Cavendish Grande Naine. When considering the complete ‘global’ samples, the mathematical programs identified *UBQ2* and *RAN* together as the most stable genes, with *α-TUB* and *ACT1pp1* as the least stable reference genes. The former pair were evaluated for normalization of RT-qPCR expression profiles in *M*. *acuminata* Calcutta 4 and *M*. *acuminata* Cavendish Grande Naine leaf samples subjected to light and dark treatments. In both *M*. *acuminata* genotypes, *RCA3* expression was down-regulated in samples from leaves covered with aluminium foil. Transcript levels of *RCA3* in the three biological replicates showed that when employing the two most stable reference genes, variation between samples was reduced, whereas the same analysis using the two least stable genes introduced considerable variation in fold change in relative expression in samples across the biological replicates (Fig. 5).

## Discussion

In this study, genes encoding proteins involved in basal cell activities in plants were evaluated as potential reference genes for normalization of gene expression data in *M*. *acuminata* leaf material through a multi-algorithm based analysis of expression stability.

Recent investigations of gene expression in *Musa* have employed numerous reference genes for normalization of RT-qPCR expression data, with focus on genotypes, tissues, development stages and response to different abiotic and biotic stresses. Reference genes employed have included Actin 1 *(ACT-1)*, clathrin adaptor complex (*CAC*), Elongation factor 1α (*EF1α*), *GAPDH*, pectate lyase (*MWPL1*), ribosomal protein 2 (*RPS2*), ribosomal protein 4 (*RPS4)*, ribosomal protein L2 (*L2*), small nuclear RNAs (*U6*), ubiquitin 2 (*UBQ2*), ubiquitin 4 (*UBQ4*) and 25 S ribosomal RNA^8,9,32–44^. Such genes, however, have often been selected based on their roles in basal cell activities, without investigation of expression stability across particular experimental conditions.

The stability of reference genes in *Musa* has so far been evaluated across a limited number of specific experimental conditions. Chen and coworkers^29^ tested candidate reference genes in Cavendish subgroup material, with stability determined across plant tissues, developmental stages, and following hormone treatment, abiotic stress and biotic stress after fruit inoculation with *Colletotrichum musae*^29^. Podevin and colleagues (2012) also evaluated expression stability of reference genes in genotypes subjected to different physiological growth conditions, osmotic stress, treatment with acetone and following biotic stress with the leaf pathogen *Pseudocercospora fijiensis* in cultivar *M*. *acuminata* Tuu Gia^30^. Zhang and colleagues^31^ also evaluated the stability of reference genes in Cavendish Grande Naine root tissues infected with *Fusarium oxysporum* f. sp. *cubense*^31^. The present work is the first describing the stability of reference genes for leaf materials during the interaction with *P*. *musae*, within the context of accurate analysis of gene expression in genotypes contrasting in resistance to the pathogen.

Multi-algorithm analysis of candidate reference gene expression stability revealed differences in the top-ranked genes observed between the results generated with each algorithm, although there was greater consistency in identification, and therefore exclusion, of the least stable candidate genes. Differences in gene stability ranking between algorithms is common^45–48^, reflecting data analysis methods for each algorithm.

In our study, combinations of stable reference genes were identified for each condition employed. When considering all samples (Global set), analyses with all the mathematical algorithms consistently revealed *UBQ2* and *RAN* ranked amongst the most stable genes, with *α-TUB* and *ACT1pp1* ranked as the least stable. Previously, Chen and co-workers^29^ also recommended *UBQ2* and *RAN* as the most stable general reference genes for Cavendish bananas. Although *UBQ* genes have been widely employed in RT-qPCR normalization across different plant species^49^, including monocot species, variation in their stability is apparent according to physiological conditions employed. In *Oryza sativa*, for example, *UBQ5* was among the most stable reference gene for plants exposed to hormone treatment, saline and drought stresses, whereas *UBQ10* was among the least stable genes observed^50^. *UBQ1* was also identified as a stable reference gene for rice plants subjected to temperature, saline and hormone treatment, fungal infection and defense signaling compounds^51,52^. In *Saccharum* sp., *UBQ1* also presented stable expression in leaf tissues^27^. *RAN* is a ubiquitously expressed gene that encodes a small soluble GTP-binding protein which plays roles in vegetative growth and stress tolerance in Arabidopsis^53^. Such high ranking of *RAN* as a reference gene has also been described in *Cucumis melo*^54^ and *Corchorus capsularis*^55^. According to Podevin and colleagues (2012), by contrast, *ACT1*, *EF1α* and *β-TUB* were identified as the most stable reference genes for leaf samples of a Cavendish genotype, with *L2* and *25 S* the least stable genes. In the same work *ACT1* and *β-TUB* were identified as the most stable genes for leaf samples inoculated with *P*. *fijiensis*^30^. If we consider our sample groups, where leaf material was inoculated with *P*. *musae*, *APT* and *GAPDH* were always among the most stable genes for Calcutta 4 samples, while *GAPDH* and *EF1α* were the most stable genes for Cavendish Grande Naine. Such results highlight how expression stability of reference genes varies according to genotype, tissue or pathogen employed in biotic stress investigation.

Primer design must also be considered in order to avoid unspecific amplification and formation of secondary structures that may hamper the polymerase chain reaction. Since *actin* is commonly used as a reference gene for RT-qPCR, we designed two primer pairs for *ACT1*, each annealing at different positions within the target gene sequence, in order to test whether they would present different stability patterns. Both primer pairs resulted in similar size amplicons, presented similar Tms (°C), and showed similar amplification efficiencies (%) (Table 1). Results revealed, however, that gene expression following amplification with primer pair *ACT1pp2* was more stable than that observed using primer pair *ACT1pp1*. Such results highlight that in addition to gene selection as reference for RT-qPCR analysis, target gene region for amplification, primer annealing temperature, and amplicon size can all influence the efficiency of an experiment^56^. In our study, we restricted amplicon size difference to a maximum of 66 bp, with all selected genes transcribed by RNA polymerase II, the same enzyme involved in gene expression of most of the target genes studied by our group. Such standardization of annealing temperature, amplicon size and consideration of RNA polymerase enzyme for reference and target gene transcription is the first for *Musa* reference genes for RT-qPCR.

RuBisCO is a key enzyme of the photosynthetic CO_2_ assimilation and photorespiratory pathways^57^. This enzyme comprises a large subunit, encoded by a plastidial gene, and smaller subunits, encoded by nuclear genes^58,59^. Activity can be regulated by RuBisCO activase (RCA), a chloroplast protein encoded by the nuclear genome. This enzyme facilitates dissociation of the sugar phosphates from RuBisCO, decreasing CO_2_ concentration for catalyzing the carbamylation of its catalytic lysine^60,61^. Previous studies have shown that RCA participates in the developmental stages of the leaves, increasing activity in mature tissues^62^. Silencing of an RCA isoform has also been shown to result in a reduction in photosynthesis^63,64^. Jiang and coworkers^65^ identified two *RCA* genes in sweet potato, where mRNA levels varied according to the intensity of light or heat^65^. Here, we identified three *RCA* genes in the *M*. *acuminata* reference (DH Pahang) genome, with *RCA3* amplifiable by conventional PCR using genomic DNA and cDNA from *M*. *acuminata* Calcutta 4 and Cavendish Grande Naine genotypes. In order to validate the stability of the reference genes, we examined the relative expression of *RCA3* in both genotypes subjected to light/dark treatments. When normalization of RT-qPCR was performed using the most stable reference genes (*UBQ2* and *RAN*), a reduced relative expression of *RCA3* was observed for dark treated leaf samples, with little variation between cDNA samples for biological replicates. By contrast, when normalization was conducted with the least stable reference genes (*α-TUB* and *ACT1pp1*), an over-estimation of transcript levels was observed in certain samples. These results clearly indicated that different reference genes influenced the relative expression levels of target genes under the specific conditions employed in this study. Similar such inaccurate estimation of relative expression of target genes during validation of potential reference genes has been shown across different plant taxa, for example the grass species *Urochloa brizantha*, and *Coffea* spp^66,67^.

In summary, this is the first report detailing validation of reference genes in *M*. *acuminata* genotypes contrasting in resistance to *P*. *musae*. As determined using the program geNorm, of the eight candidate reference genes tested, a minimum number of two genes were always sufficient to provide the necessary accuracy for RT-qPCR analysis under the experimental conditions employed. Considering the frequency of the top ranked stably expressed reference genes, *UBQ2* and *RAN* are appropriate for normalization in studies that consider *P*. *musae*-inoculated or non-inoculated leaf samples for both *M*. *acuminata* genotypes Calcutta 4 and Cavendish Grande Naine. The various reference gene sets developed here for the genotypes contrasting in resistance to the Sigatoka leaf spot pathogen *P*. *musae* will enable accurate analysis of gene expression and pathways activated in this pathosystem, advancing understanding of host responses to this important foliar pathogen.

## Methods

### Plant material

*M*. *acuminata* plantlets of susceptible commercial Cavendish subgroup cultivar ‘Grande Naine’ (CAV) (AAA) (Musa International Transit Centre accession ITC0654) and the resistant wild fertile diploid Calcutta 4 (C4) (AA) (accession ITC0249), widely employed as a breeding genitor genotype, were provided by Embrapa Cassava and Tropical Fruits. *In vitro-derived* plantlets were grown in a mix of sterilized substrate of soil and sand (1:1), fertilizer and lime under glasshouse conditions with a 12-h light/12-h dark photoperiod, 85% relative humidity and an average temperature of 25 °C.

### Leaf inoculation with *P*. *musae*

The youngest emerged leaf from 3 month old plants was artificially inoculated with a suspension comprising 2 × 10^4^ conidiospores ml^−1^ of *P*. *musae* and the surfactant Tween 20 at 0.05%. Non-inoculated control plants were inoculated with only the water-surfactant mixture. A total of three independent replicates were collected for each sample.

### RNA extraction and cDNA synthesis

Leaf material (2.5 cm^2^ area) from pathogen-inoculated and non-inoculated controls was collected at 3 and 12 days, flash frozen in liquid nitrogen and stored at −80° C. Total RNA was extracted from leaf tissues using the Concert^®^ Plant RNA Reagent (Invitrogen, Carlsbad, CA, USA), with purification using the INVISORB Spin Plant RNA Mini Kit (Invitek, Hayward, CA, USA), according to manufacturer’s instructions. A total of 1 μg of each total RNA was treated with 2 U of DNase I (New England Biolabs, Ipswich, MA., USA) for elimination of residual DNA. Analysis of total RNA concentration and integrity was conducted following 1% agarose gel electrophoresis and Nanodrop ND-1000 spectrophotometry (Thermo Fischer Scientific, Waltham, MA, USA).

For cDNA synthesis, pools containing equimolar amounts of total RNA from three biological replicates were prepared. In total, eight pools were prepared, one for each experimental condition (Table 1). A total of 1 μg of total RNA was reverse transcribed to cDNA using Super Script II RT and Oligo(dT)_16–18_ primers (Invitrogen, Carlsbad, CA, USA).

### Primer design

For the design of potential reference genes with stable expression in *M*. *acuminata* leaf tissues, candidate genes were selected that encode proteins involved in basal cell activites in diverse plant species, including *Musa* (Table 1). OligoPerfect^TM^ Designer (Thermo Fischer Scientific, Waltham, MA, USA) was employed for design of specific primers for each candidate reference gene. Expected target amplicons varied from 80 to 150 bp, with all primers designed to specifically amplify predicted exon-exon junctions, guaranteeing amplification from cDNA rather than potential contaminant genomic DNA. Primer specificity was initially tested by electronic PCR against a local database of RNAseq data for *Musa* and against the reference whole genome sequence for *M*. *acuminata*, genotype DH Pahang at Phytozome (phytozome.jgi.doe.gov)^10^. Specificity and efficiency was subsequently tested against cDNA originating from leaf tissues of *M*. *acuminata* CAV and C4 (data not shown). Primer sequences that resulted in specific amplification are listed in Table 1.

### Real-time quantitative PCR

RT-qPCR analysis of expression of candidate reference genes was conducted using a Platinum SYBR Green qPCR Super Mix-UDG w/ROX kit (Invitrogen, Carlsbad, CA, USA). PCR amplifications were performed on an ABI StepOne^®^ Real-Time PCR thermocycler (Applied Biosystems, Foster City, USA), using three independent experimental replicates and three technical replicates per amplification. For PCR reactions, mixtures contained 2 μL of a 1:20 dilution of template cDNA, 0.2 μM of each primer (Table 1) and 5 μL Platinum^®^ SYBR^®^ Green qPCR Super Mix-UDG w/ROX kit (Invitrogen, Carlsbad, CA, USA), to a final volume of 10 μL. PCR amplification was conducted using an initial step of 52 °C for 2 min, 95 °C for 10 min, followed by 40 cycles of denaturation at 95 °C for 15 s, primer annealing and extension at 60 °C for 60 s. Primer specificity was verified by analyzing the Tm (dissociation) of amplified products using SDS 2.2.2 software (Applied Biosystems, Foster City, USA).

### Baseline correction and efficiency determination

Raw ΔRn data was analysed using the LinRegPCR program, version 2017.1, with all calculations performed according to the manufacturer’s instructions. Baseline fluorescence was corrected by reconstruction of the log-linear phase. Data was then grouped and applied to determine RT-qPCR mean efficiency for each gene. The same program was subsequently used to calculate the average quantification cycles (Cqs) per sample^68^.

### Expression stability and relative expression analysis

Expression data for each candidate reference gene was visualized as a quantification cycle (Cq) value from each RNA sample, as obtained from LinRegPCR analysis. This value represents the number of amplification cycles required to reach a default threshold value for detection during the exponential amplification phase of the PCR reaction.

The expression stability of each candidate reference gene was determined using the algorithms geNorm^22^, NormFinder^23^ and BestKeeper^24^, according to the default software parameters. GeNorm and NormFinder use the Cq data to calculate Relative Quantities (RQ) by application of the formula RQ = 2^(ddCq)^. GeNorm evaluates expression stability by calculating an average M-value, which is defined as the pairwise variation of one gene against all other genes. Those genes presenting the lowest M-values below a threshold of 0.5 have the most stable expression. The program also calculates a V-value, a pairwise variation (Vn/n + 1) between each reference gene, to determine the optimal number of genes required for normalization. In the case of NormFinder, variation between groups of genes at both the inter- and intra-group level is calculated with ANOVA and used to generate a stability value (SV) for each reference gene, with the most stable genes presenting the lowest SV values below the default limit of 0.5. As such, this method can be more appropriate for providing estimates of expression variation from subsets of different sample types^23^. BestKeeper is a tool that calculates the geometric mean of Cq values, with the most stable genes indicated by high correlation coefficients and low standard deviations. Raw Cq values are used to calculate the geometric mean and rank the reference genes according to the lowest stability values. A global re-ranking of the candidate genes stability was conducted using the web-based tool RefFinder (http://www.leonxie.com/referencegene.php). RefFinder allows integration of data generated with all the algorithms geNorm, NormFinder, Bestkeeper and delta Ct method, without considering RT-qPCR efficiencies. Through assigning a weight to each individual gene, a geometric mean weight of the candidate genes can be calculated, which can then be used to rank the stability of each individual reference gene.

### *RCA3* expression analysis

The youngest fully emerged leaf from 3 month old plants was subjected to a photoperiod of 12/12 (L/D) at 25 °C for 7 days. In a second group, leaves were covered in aluminium foil paper and maintained at 25 °C for 7 days. After this period, leaves were flash frozen in liquid nitrogen and preserved at −80 °C until use. Three independent replicates were collected for each leaf sample. Total RNA, cDNA synthesis and real time PCR were performed as described previously.

## Supplementary information

Dataset 1

Dataset 2

## Data Availability

All data generated and analyzed during the study is included in the published article and its Supplementary Information files.
